# Inhibition of P-Glycoprotein and Multidrug Resistance-Associated Protein 2 Regulates the Hepatobiliary Excretion and Plasma Exposure of Thienorphine and Its Glucuronide Conjugate

**DOI:** 10.3389/fphar.2016.00242

**Published:** 2016-08-09

**Authors:** Ling-Lei Kong, Guo-Lin Shen, Zhi-Yuan Wang, Xiao-Mei Zhuang, Wei-Bin Xiao, Mei Yuan, Ze-Hui Gong, Hua Li

**Affiliations:** ^1^State Key Laboratory of Toxicology and Medical Countermeasures, Beijing Institute of Pharmacology and ToxicologyBeijing, China; ^2^Beijing Key Laboratory of Drug Targets Identification and Drug Screening, Centre for Pharmaceutical Screening, Institute of Materia Medica, Chinese Academy of Medical Sciences, Peking Union Medical CollegeBeijing, China; ^3^Research Center for Import-Export Chemicals Safety of General Administration of Quality Supervision, Inspection and Quarantine of People's Republic of China, Chinese Academy of Inspection and QuarantineBeijing, China

**Keywords:** thienorphine, multidrug resistance-associated protein 2, P-glycoprotein, biliary excretion, siRNA, drug interaction

## Abstract

Thienorphine (TNP) is a novel partial opioid agonist that has completed phase II clinical evaluation as a promising drug candidate for the treatment of opioid dependence. Previous studies have shown that TNP and its glucuronide conjugate (TNP-G) undergo significant bile excretion. The purpose of this study was to investigate the roles of efflux transporters in regulating biliary excretion and plasma exposure of TNP and TNP-G. An ATPase assay suggested that TNP and TNP-G were substrates of P-gp and MRP2, respectively. The *in vitro* data from rat hepatocytes showed that bile excretion of TNP and TNP-G was regulated by the P-gp and MRP2 modulators. The accumulation of TNP and TNP-G in HepG2 cells significantly increased by the treatment of mdr1a or MRP2 siRNA for P-gp or MRP2 modulation. In intact rats, the bile excretion, and pharmacokinetic profiles of TNP and TNP-G were remarkably changed with tariquidar and probenecid pretreatment, respectively. Tariquidar increased the C_max_ and AUC_0-*t*_ and decreased MRT and T_1/2_ of TNP, whereas probenecid decreased the plasma exposure of TNP-G and increased its T_1/2_. Knockdown P-gp and MRP2 function using siRNA significantly increased the plasma exposure of TNP and TNP-G and reduced their mean retention time in mice. These results indicated the important roles of P-gp and MRP2 in hepatobiliary excretion and plasma exposure of TNP and TNP-G. Inhibition of the efflux transporters may affect the pharmacokinetics of TNP and result in a drug-drug interaction between TNP and the concomitant transporter inhibitor or inducer in clinic.

## Introduction

Opioid abuse and dependence remains a serious worldwide health problem. Opioid partial agonists have been proven to be effective in reducing illicit opioid use and exhibit a good safety profile, particularly with respect to lowering the respiratory depression, and dependence potential when compared to the full μ-agonist. Thienorphine (TNP), N-Cyclopropylmehtyl-7α-[(R)-1-hydroxyl-1-methyl-3-(thien-2-yl)propyl]-6, 14-endoethanotetrahydronooripavine, was synthesized as a ramification of buprenorphine, and identified to be a novel non-selective opioid partial agonist (Liu et al., 2005; Yu et al., 2006). When compared with buprenorphine, TNP showed high binding affinity with the μ-opioid receptor *in vitro* and a better oral bioavailability *in vivo* (Li et al., 2007; Yu et al., 2013). TNP was developed for the treatment of opioid dependence. Recently, the phase II clinical trial was completed, and the result was positive.

The absorption, distribution, metabolism, and excretion of TNP have been studied in pre-clinical animals (Kong et al., 2007). TNP was extensively metabolized in the liver and gut to form a number of oxidative and conjugated metabolites. Glucuronide conjugate (TNP-G) was the major active metabolite in the circulation of both rat and dog models, with weaker pharmacological effects than TNP. TNP underwent a significant biliary clearance in rats with nearly 24% of the oral dose excreted from bile within 24 h. Excreted TNP and TNP-G via bile could be reabsorbed rapidly from the rat intestine to form enterohepatic circulation (EHC), which was confirmed using a paired rat model and *in situ* perfused rat intestinal preparations (Deng et al., 2010). EHC may prolong the pharmacologic effect of a compound by maintaining the therapeutic concentrations for an extended period of time (Bi et al., 2006; Shitara and Sugiyama, 2006). The significant EHC of TNP and TNP-G was believed to be one of the main contributors to the long-acting effect of TNP.

Drug transporters are membrane proteins that have a major impact on the absorption, distribution, and elimination of a wide range of drugs. They are distributed and expressed in many tissues including the intestine, liver, kidney, and brain. In particular, hepatic drug transporters contribute significantly to the hepatic exposure, and biliary excretion of various endogenous and exogenous compounds (Tchaparian et al., 2011; Kong et al., 2015a). P-glycoprotein (P-gp), multidrug resistance-associated protein 2 (MRP2) and breast cancer resistance protein (BCRP) are hepatic efflux transporters responsible for the hepatobiliary excretion of drugs. They extrude substrates into the bile and restrict the (re)uptake of substrates from the gut (Sai, 2005). Preclinical and clinical studies have demonstrated that inhibition or induction of these transporters may influence the clearance and pharmacokinetics of drugs and lead to altered toxicity or therapeutic efficacy (Zhuang et al., 2013; Kong et al., 2015a). Therefore, it has become critically important to characterize a drug candidate as a substrate or regulator of transporters during drug development (Giacomini et al., 2010). As a promising agent for treatment of opioid abuse, TNP has a great chance for coadministration with other clinical drugs, such as anti-virus drugs, and anti-inflammatory drugs. Some of these drugs are known substrates or regulators of drug enzymes or transporters (Kimoto et al., 2012). Concomitant use of TNP with these drugs may result in drug-drug interactions (DDIs).

The membrane transport of TNP was previously investigated using a Caco-2 monolayer model, and the results suggested that efflux of TNP might associate with the active transport process (Li et al., 2010). A screening study using an ATPase assay indicated that TNP and TNP-G were the substrates of P-gp and MRP2, respectively. Since substantial portion of TNP excreted from bile in rats, it is speculated that canalicular efflux may be involved in the biliary excretion of TNP and its metabolites. Based on the fact that biliary excretion and EHC contribute remarkably to the prolonged pharmacological activity, it is necessary to investigate the roles of efflux transporters in the pharmacokinetics of TNP and assess the DDI potential of transporter modulation. In the present study, the effects of transporter modulators on biliary excretion and pharmacokinetics of TNP and TNP-G were determined in sandwich-cultured rat hepatocytes (SCRH), bile-duct cannulated (BDC), and intact rats. Chemically synthesized small interfering RNA (siRNA) was also used to specifically knockdown P-gp or MRP2 gene expression in Human liver hepatocellular carcinoma cells (HepG2) and mice to further assess the role of transporters on hepatobiliary elimination of TNP and its metabolites.

## Materials and methods

### Materials

**Figure 1:**
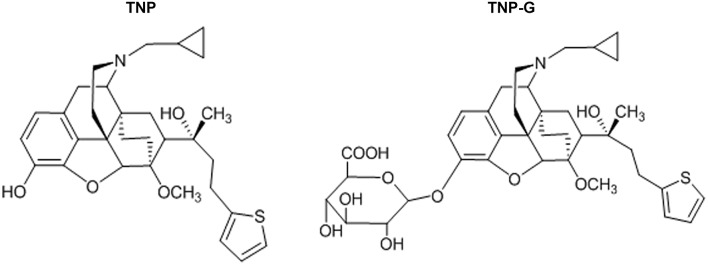
**The chemical structure of TNP and TNP-G**.

Thienorphine hydrochloride (99.5% in purity, Figure 1) and thienorphine-3-glucuronide (99% in purity, Figure 1) were synthesized using the chemical synthesis laboratory of the Institute. Tariquidar, propranolol, verapamil, probenecid, quercetin, dexamethasone, methotrexate, and collagenase were purchased from Sigma (St. Louis, MO, USA). MDR1, MRP2 and BCRP membranes as well as an ATPase assay kit were purchased from BD Gentest Co. (Woburn, MA). Acetonitrile was of HPLC grade (J&K Chemical LTD), and other reagents were all of an analytical grade.

### Animals

Male SD rats (weight, 220–260 g) and male BALB/C mice (weight, 18–22 g) were supplied by the Beijing Experimental Animal Center (Beijing China). All animals were maintained on a 12 h light/dark cycle with free access to water and lab chow. All of the animal experiments were conducted at the Beijing Center for Drug Safety Evaluation following the protocol of the Institutional Animal Care and Use Committee of the Center.

### ATPase screening assay

The ATPase assay was performed using an ATPase assay kit following the instruction manual. Verapamil, probenecid, and sulfasalazine were tested as model substrates of MDR1, MRP2, and BCRP, respectively. The stimulated ATPase activities were calculated by dividing the amount of inorganic phosphate produced by the incubation time and the amount of protein used in the incubation.

### Culture of rat hepatocytes

Rat hepatocytes were isolated with a two-step perfusion method as reported previously (Shen et al., 2014). Hepatocyte suspensions were added to the precoated dishes at a density of 0.5 × 10^6^ cells/well on 24-well plates and allowed to attach for 4 h, at which time the medium was aspirated to remove any unattached cells and fresh medium was added. Twenty-four hours later, cells were overlaid with BD Matrigel at a concentration of 0.25 mg ml^−1^ in 0.5 ml of ice-cold DMEM-F12. The hepatocytes were cultured for 4 days, and the medium was changed daily.

### Bile clearance study in SCRH

On day 5, SCRH were rinsed twice and then preincubated for 30 min at 37°C with 0.5 ml of warmed Hanks' balanced salt solution (HBSS) containing Ca^2+^/Mg^2+^ (standard buffer) to maintain or Ca^2+^/Mg^2+^-free buffer to disrupt the tight junctions sealing the bile canalicular networks. For the bile clearance study, the SCRH were incubated with 20 μM TNP in HBSS at 37°C for 2, 5, 10, 20, 30, or 60 min or with 20 μM TNP-G for 5–120 min. To investigate the effect of inhibition on bile clearance, the P-gp inhibitors, verapamil (10, 50, or 100 μM) or tariquidar (0.5, 2.5, or 5 μM), or MRP2 inhibitors, probenecid (10, 50, or 100 μM) or methotrexate (10, 50, or 100 μM), were preincubated for 30 min with the SCRH prior to the bile clearance study. To assess the effect of induction, on day 3 to day 5 the SCRH was replaced with fresh culture media containing the P-gp inducer quercetin (3, 15, or 30 μM), or MRP2 inducer dexamethasone (10, 50, or 100 μM) every 24 h. After co-incubation with TNP or TNP-G for 60 min, the supernatant was aspirated from the cells and uptake was stopped by washing the cells three times with ice-cold HBSS. The cells were then lysed and stored at −80°C. The samples were analyzed using liquid chromatography coupled with tandem mass spectrometry (LC-MS/MS). The total protein concentration of cell lysates was quantified using the BCA assay.

### Cell culture and uptake experiment in HepG2 cells transfected with siRNA

HepG2 cells were obtained from the ATCC and maintained in DMEM that was supplemented with 10% fetal bovine serum, penicillin (100 U/ml) and streptomycin (100 μg/ml). Cells were seeded into 24-well plates at a density of 1 × 10^5^ cells/well and cultured for 24 h. Transfection was performed in Opti-MEM (Invitrogen, Carlsbad, CA, USA) with synthetic siRNA (80 pM) by using Lipofectamine 2000 (Invitrogen, Carlsbad, CA, USA) for 6 h, and the medium was replaced with fresh DMEM. This condition was optimized in our previous study based on the transfection efficiency and cellular viability (Kong et al., 2015b). Following incubation for 48 h, the cells were washed three times with phosphate-buffered saline buffer (PBS), and then 20 μM TNP, or TNP-G was added to the DMEM and incubated for 5, 15, 30, or 60 min at 37°C. After incubation, the supernatant was aspirated from the cells, and the uptake was stopped by washing the cells three times with ice-cold PBS. The cells were lysed and stored at −80°C. The samples were analyzed using liquid chromatography coupled with tandem mass spectrometry (LC-MS/MS).

### Biliary excretion and pharmacokinetic studies in rats

Bile-duct cannulated (BDC) rats were surgically prepared as previously reported. The BDC rats were divided into three groups. The control group was intravenously injected with saline 15 min prior to an intragastric dose of TNP (12 mg/kg). The two treatment groups were intravenously given probenecid (200 mg/kg) or tariquidar (10 mg/kg) 15 min prior to the TNP oral dose. The bile samples were collected at the time intervals of 0–1, 1–2, 2–4, 4–6, 6–8, 8–12, 12–24, 24–36, 36–48, and 48–60 h. The pharmacokinetic study was performed in intact rats following the same dose above. Blood samples (200 μl) were collected at 5, 15, and 30 min and 1, 2, 4, 6, 8, 12, and 24 h post-TNP dose. The plasma was separated and stored at −80°C until analysis.

### Pharmacokinetics of TNP and its metabolites in mice treated with siRNA

The pharmacokinetic study in mice treated with siRNA was conducted according to our previously reported study (Kong et al., 2015a). Mice were divided into three groups to collect the blood samples: (1) TNP + NC-siRNA group; (2) TNP + mdr1a siRNA group; (3) TNP + MRP2 siRNA group. The siRNA treatment groups were intravenously injected with NC-siRNA, MRP2 siRNA, or mdr1a siRNA 2 days prior to receiving the TNP oral dose. Blood samples were collected at 5, 15, and 30 min and 1, 2, 4, 6, 8, and 12 h after the TNP dosing. The plasma was separated and stored at −80°C until analysis.

### LC-MS/MS analysis of TNP and TNP-G

The concentration of TNP and TNP-G in cells, plasma and bile samples were analyzed according to a previously reported method (Kong et al., 2007). An Aglient 6410B triple quadrupole mass spectrometer equipped with the Agilent1290 Infinity UHPLC system (Agilent Technologies, Santa Clara, CA) was used. TNP, TNP-G, and propranolol (an internal standard) were eluted from an Agilent C18 column with a mobile phase consisting of equal volumes of 0.1% formic acid in a 5 mM ammonium formate solution and 0.1% formic acid in acetonitrile. Quantification was performed using selected reaction monitoring with the transitions of m/z 522-m/z 97, m/z 698-m/z 522.4, and m/z 260-m/z 116.2 for TNP, TNP-G, and propranolol, respectively. The samples were pretreated with 3-fold volume of methanol-acetonitrile (1:1, v/v), containing 20 ng ml^−1^ propranolol to precipitate the proteins and then centrifuged at 4°C at 14,000 × g for 10 min. The supernatant (10 μl) was injected into the LC-MS/MS for analysis. The lower limit of quantification was 0.1 ng/ml for TNP, and TNP-G, and the standard curves ranged from 0.1 to 1000 ng.

### Data analysis

The biliary excretion index (BEI) and *in vitro* intrinsic biliary clearance (CL_bile, int_, ml/min/kg) were calculated based on the published equations (Liu et al., 1999b):

(1) $$\begin{array}{ll} \text{BEI}= & \left({\text{Accumulation}}_{\text{cell }+\text{ bile}}-{\text{Accumulation}}_{\text{cells}}\right)\\ /{\text{Accumulation}}_{\text{cell }+\text{ bile}}\times 100\% \end{array}$$

(2) $$\begin{array}{ll} {\text{CL}}_{\text{bile},\text{ int}}= & \left(\text{Accumulatio}{\text{n}}_{\text{standard}}-{\text{Accumulation}}_{{\text{Ca}}^{2+}{/\text{Mg}}^{2+}\text{-free}}\right)\\ /\text{incubation time}\times \text{concentration }\left(\text{medium}\right)\\ \times \;200\times 40 \end{array}$$

Drug concentrations in the medium were defined as the initial substrate concentration in the incubation medium. The CL_bile, int_ was scaled to kilograms of body weight assuming the following: 200 mg protein g^−1^ rat liver tissue and 40 g rat liver tissue kg^−1^ body weight (Liu et al., 1999a).

The predicted *in vivo* CL_bile, pred_ values were estimated according to the equations below (Nakakariya et al., 2012):

(3) $$\begin{array}{l} {\text{CL}}_{\text{bile},\text{ pred}}=\text{Qp }\times \;in\;vitro\;{\text{CL}}_{\text{bile},\text{ int}}/\left(\text{Qp }+\;in\;vitro\;{\text{CL}}_{\text{bile},\text{ int}}\right) \end{array}$$

where Qp represents the hepatic plasma flow rate (40 ml/min/kg). In Equation (3), the plasma unbound fraction (f_u, p_) was assumed to be unity. Taking into consideration the unbound fraction, the following Equation (4) was applied:

(4) $$\begin{array}{ll} {\text{CL}}_{\text{bile},\text{ pred}}=\text{Qp}\times {\text{f}}_{\text{u},\text{ p}}\times in\;vitro\;{\text{CL}}_{\text{bile},\text{ int}}\;\\ \;/\left(\text{Qp}+{\text{f}}_{\text{u},\text{ p}}\times in\;vitro\;{\text{CL}}_{\text{bile},\text{ int}}\right)\; & \; \end{array}$$

The observed *in vivo* biliary clearance (CL_bile, obs_, ml/min/kg) was calculated according to Equation 5:

(5) $$\begin{array}{l} {\text{CL}}_{\text{bile},\text{ obs}}={\text{Bile Accumulation Amount}}_{0–24\text{h}}/{\text{AUC}}_{0–24\text{h}} \end{array}$$

where Amount_0–24h_ represents the cumulative amount of compounds recovered in the bile from 0 to 24 h, and AUC_0–24h_ represents the area under the plasma concentration-time curve from 0 to 24 h.

All of the data were expressed as the mean ± SD. Differences between groups were compared by Student's *t*-test for analysis of unpaired data or one-way ANOVA. The statistical significance was accepted at *P* < 0.05.

## Results

### Identification of efflux transporters associated with TNP and TNP-G

To identify the transporters responsible for TNP and TNP-G biliary efflux, the ATPase screening assay was conducted. The results (Table 1) demonstrated that the ATPase activity of the MDR1 membrane was significantly increased by both verapamil and TNP (from 4.3 ± 0.9 to 31.8 ± 0.7, and 19.3 ± 0.3 nmol/min/mg), but not TNP-G. The ATPase activity of the MRP2-expressing membrane was significantly increased by probenecid and TNP-G (from 1.2 ± 0.5 to 5.7 ± 0.2 and 4.1 ± 0.6 nmol/min/mg). The model substrate sulfasalazine enhanced the ATPase activity in the BCRP study, but this was not observed for either TNP or TNP-G incubation. The results indicated that TNP was a substrate of MDR1 and that TNP-G was a MRP2 substrate. BCRP may not be involved in the efflux clearance of TNP and TNP-G.

**Table 1:** **Identification of efflux transporters associated with TNP and TNP-G**.

**Vanadate-sensitive ATPase activities (nmol/min/mg)**					
**Compound**	**MDR1**	**Compound**	**MRP2**	**Compound**	**BCRP**
Blank	4.3 ± 0.9	Blank	1.2 ± 0.5	Blank	6.4 ± 1.7
Verapamil	31.8 ± 0.7^##^	Probenecid	5.7 ± 0.2^##^	Sulfasalazine	32.2 ± 1.3^##^
TNP	19.3 ± 0.3^##^	TNP	0.8 ± 0.2	TNP	5.9 ± 0.8
TNP-Glu	4.2 ± 0.8	TNP-Glu	4.1 ± 0.6^##^	TNP-Glu	5.8 ± 0.8

MDR1, MRP2, or BCRP membranes and TNP or TNP-G (1 mM for MDR1, and BCRP, 30 mM for MRP2) were pre-incubated for 5 min (MDR1 and MRP2), or 10 min (BCRP). The reactions were started by addition of 50 mM MgATP, followed by incubation at 37°C for 20 min for MDR1, 40 min for MRP2, or 30 min for BCRP. Verapamil (1 mM), probenecid (100 mM), and sulfasalazine (1 mM) were as model substrates, respectively. Data are expressed as mean ± SD (n = 3).

## *P < 0.01 compared with blank*.

### Cell accumulation and biliary excretion of TNP and TNP-G in SCRH

**Figure 2:**
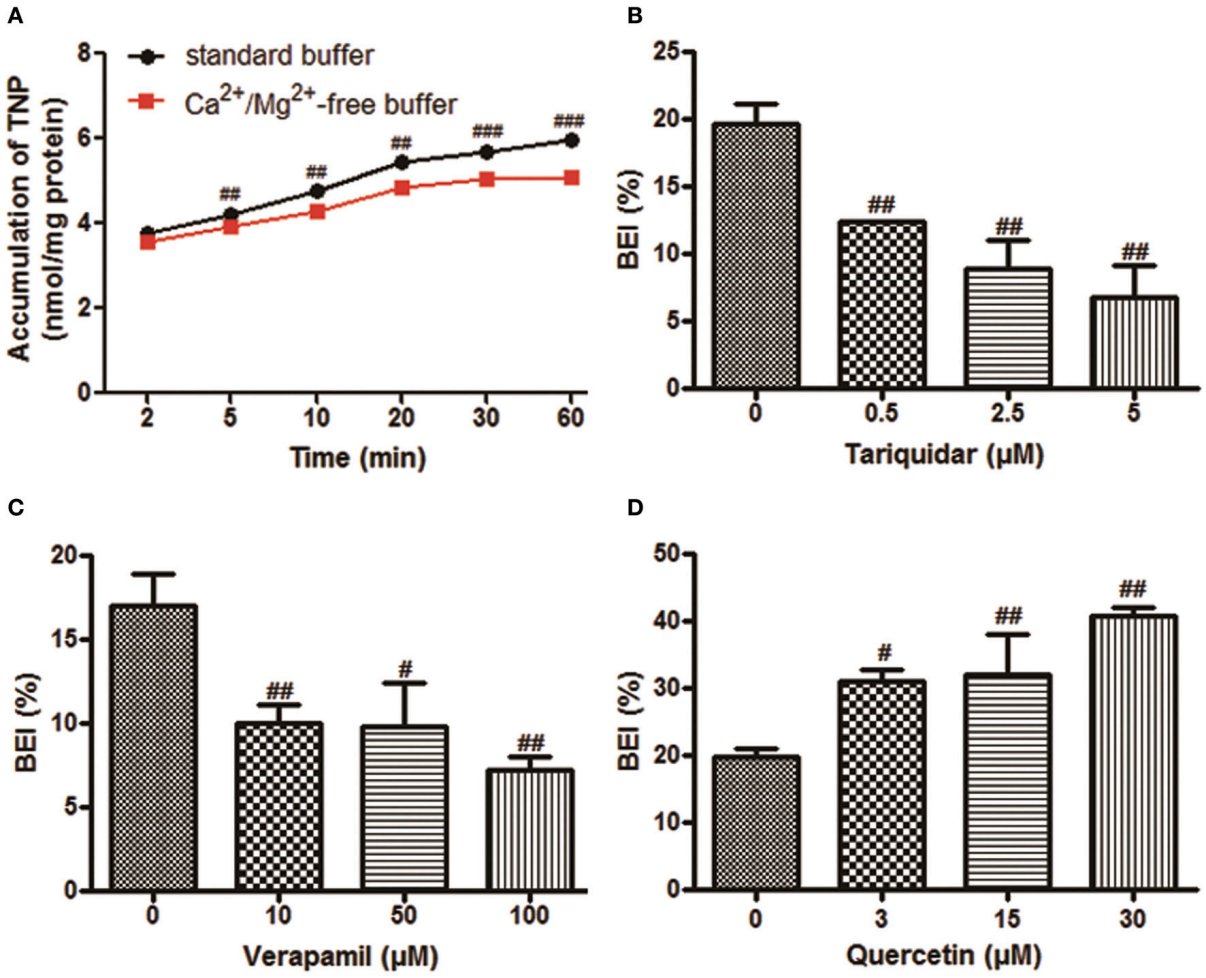
**The biliary excretion of TNP in SCRH**. TNP (20 μM) was added after pre-incubation of SCRH with standard buffer or Ca^2+^/Mg^2+^-free buffer for 30 min to investigate the accumulation of TNP in SCRH. The inhibitors were pre-incubated with SCRH for 30 min, and the inducer was pre-incubated with SCRH for 3 days before being co-incubated with TNP (20 μM) for an additional 60 min. Samples were collected at different time points. Data are presented as the mean ± SD (*n* = 3). ^#^*P* < 0.05, ^*##*^*P* < 0.01, ^*###*^*P* < 0.001 compared with the control group. **(A)** The accumulation of TNP in SCRH; **(B)** The effect of tariquidar on BEI of TNP; **(C)** The effect of verapamil on BEI of TNP; **(D)** The effect of quercetin on BEI of TNP.

**Figure 3:**
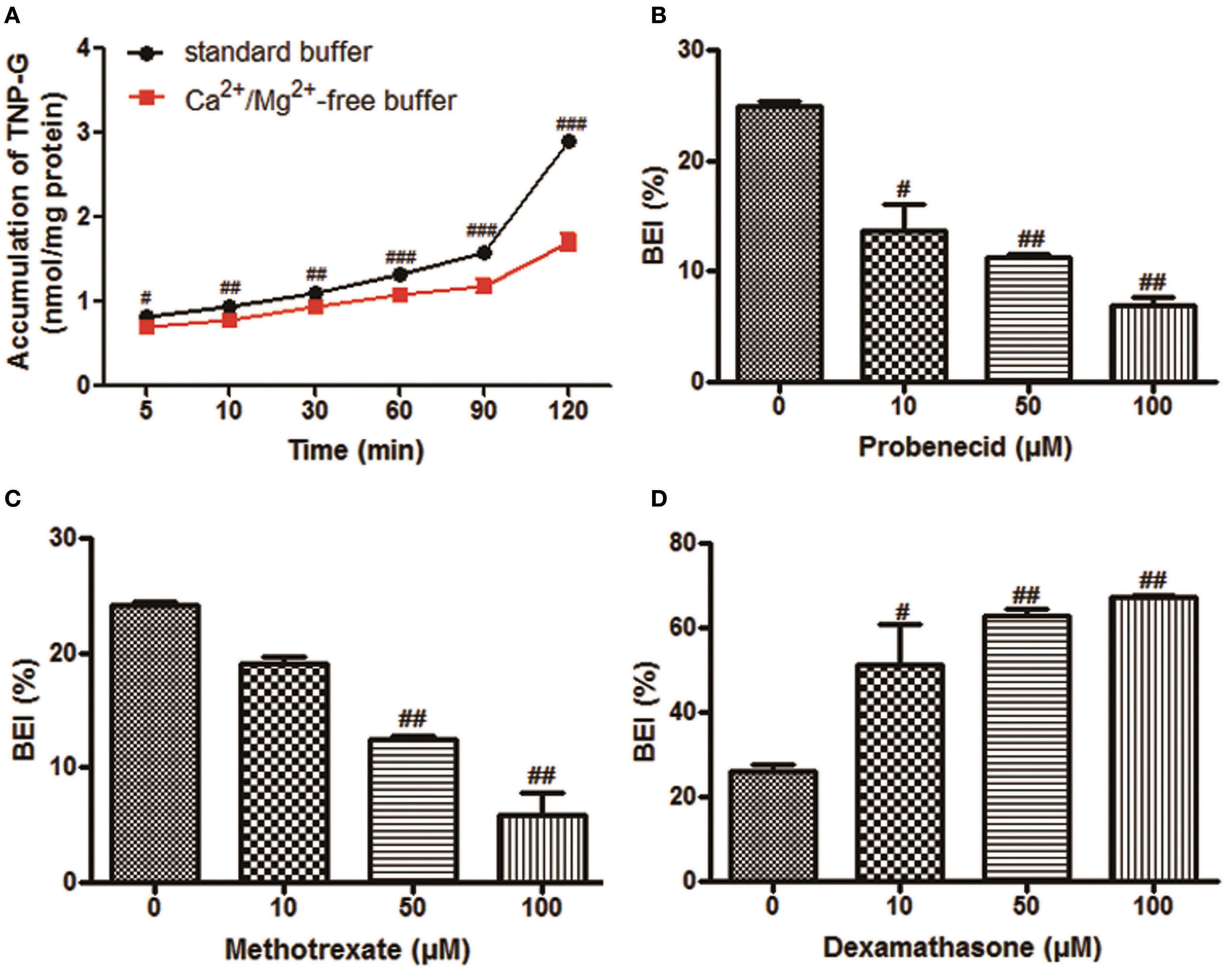
**The biliary excretion of TNP-G in SCRH**. TNP-G (20 μM) was added after pre-incubation of SCRH with standard buffer or Ca^2+^/Mg^2+^-free buffer for 30 min to investigate the accumulation of TNP-G in SCRH. The inhibitors were pre-incubated with SCRH for 30 min, and the inducer was pre-incubated with SCRH for 3 days before being co-incubated with TNP-G (20 μM) for an additional 60 min. Data are presented as the mean ± SD (*n* = 3). ^#^*P* < 0.05, ^*##*^*P* < 0.01, ^*###*^*P* < 0.001 compared with the control group. **(A)** The accumulation of TNP-G in SCRH; **(B)** The effect of probenecid on BEI of TNP-G; **(C)** The effect of methotrexate on BEI of TNP-G; **(D)** The effect of dexamethasone on BEI of TNP-G.

The time-dependent intracellular accumulation of TNP and TNP-G in SCRH incubated in standard HBSS or Ca^2+^/Mg^2+^-free buffer was shown in Figures 2A, 3A. At each time point, the cell accumulation in the standard buffer (cell+bile) was significantly higher than that in Ca^2+^/Mg^2+^-free HBSS (cells only), indicating excretion of TNP and TNP-G in the bile canalicular network. The average BEI for TNP after a 60 min incubation was 14.5 ± 0.9 and 41.4 ± 2.6 for TNP-G after a 120-min incubation. The CL_bile, int_ of TNP and TNP-G was 5.8 ± 0.5 and 11.2 ± 2.7 ml/min/kg (Supplementary Table 1). The *in vivo* CL_bile, pred_ values were predicted, based on the total concentration (Equation 3) or the plasma unbound fraction (Equation 4), to be 5.0 ± 0.4, or 0.3 ± 0.02 ml/min/kg for TNP and 8.7 ± 1.7 or 0.2 ± 0.01 ml/min/kg for TNP-G (Supplementary Table 1).

### P-gp mediated TNP bile clearance in SCRH

Based on the ATPase assay results, known P-gp inhibitors and an inducer were chosen to further assess the role of this transporter in the TNP canalicular efflux in SCRH. Figures 2B,C show that the known P-gp inhibitors (tariquidar and verapamil) could reduce the BEI of TNP in a dose-dependent manner. Tariquidar (5 μM) and verapamil (100 μM) significantly decreased the BEI to 34.5 and 42.4% of the control cells. The CL_bile, int_ of TNP was reduced to 83.1 and 73.7% by tariquidar and verapamil, respectively, (Supplementary Table 2). After pretreatment for 3 days with a dose of 30 μM quercetin, a P-gp inducer, the BEI, and CL_bile, int_ of TNPincreased to 2.1- and 1.7-fold compared with the control (Figure 2D, Supplementary Table 2).

### MRP2 mediated bile excretion of TNP-G in SCRH

As shown in Figures 3B,C, dose-dependent inhibition of biliary excretion of TNP-G was observed after pretreatment with the inhibitors. At the concentration of 100 μM, probenecid and methotrexate remarkably reduced the BEI to 22.8 and 20% of the control cells. The CL_bile, int_ was decreased by 78.1 and 74.2% at the same inhibitory concentration. When pre-incubated for 3 days with dexamethasone (100 μM), a MRP2 inducer, the BEI, and CL_bile, int_of TNP-G were increased up to 2.6- and 2.7-fold (Figure 3D, Supplementary Table 3).

### Effects of siRNA on the intracellular concentration of TNP and TNP-G in HepG2 cells

**Figure 4:**
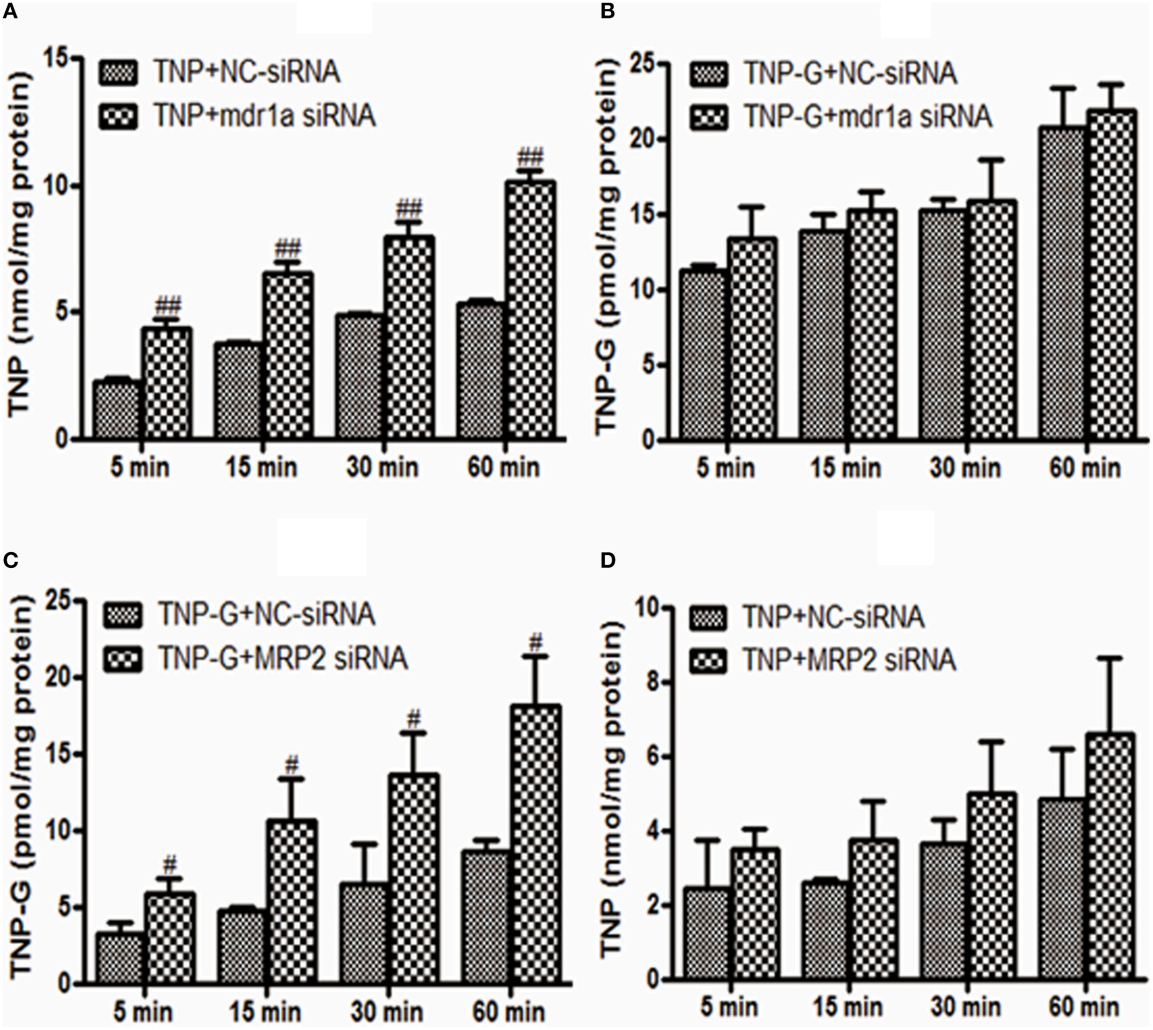
**Effects of siRNA on TNP and TNP-G accumulation in HepG-2 cells**. HepG-2 cells were transfected with siRNAs. After 48 h, TNP, or TNP-G (20 μM) was added, and samples were collected at different time points. Data are presented as the mean ± SD (*n* = 3). ^#^*P* < 0.05, ^*##*^*P* < 0.01 compared with the NC-siRNA group. **(A)** Effect of mdr1a siRNA on TNP accumulation; **(B)** Effect of mdr1a siRNA on TNP-G accumulation; **(C)** Effect of MRP2 siRNA on TNP-G accumulation; **(D)** Effect of MRP2 siRNA on TNP accumulation.

To further investigate the roles of P-gp or MRP2 in the cellular uptake of TNP and TNP-G *in vitro*, mdr1a siRNA, or MRP2 siRNA was used to knock down the transporter expression in HepG2 cells. As shown in Figure 4, the intracellular concentration of TNP and TNP-G increased in a time-dependent manner. Compared with the NC-siRNA group, the efflux of TNP and TNP-G were significantly inhibited by mdr1a siRNA and MRP2 siRNA, respectively, and the intracellular concentrations were increased 1.9- and 2.1-fold. In addition, the intracellular concentration of TNP-G and TNP was not impacted by mdr1a siRNA and MRP2 siRNA, respectively. This result further indicated that P-gp and MRP2 play a role in transportation of TNP or TNP-G.

### Biliary excretion in BDC rats

**Figure 5:**
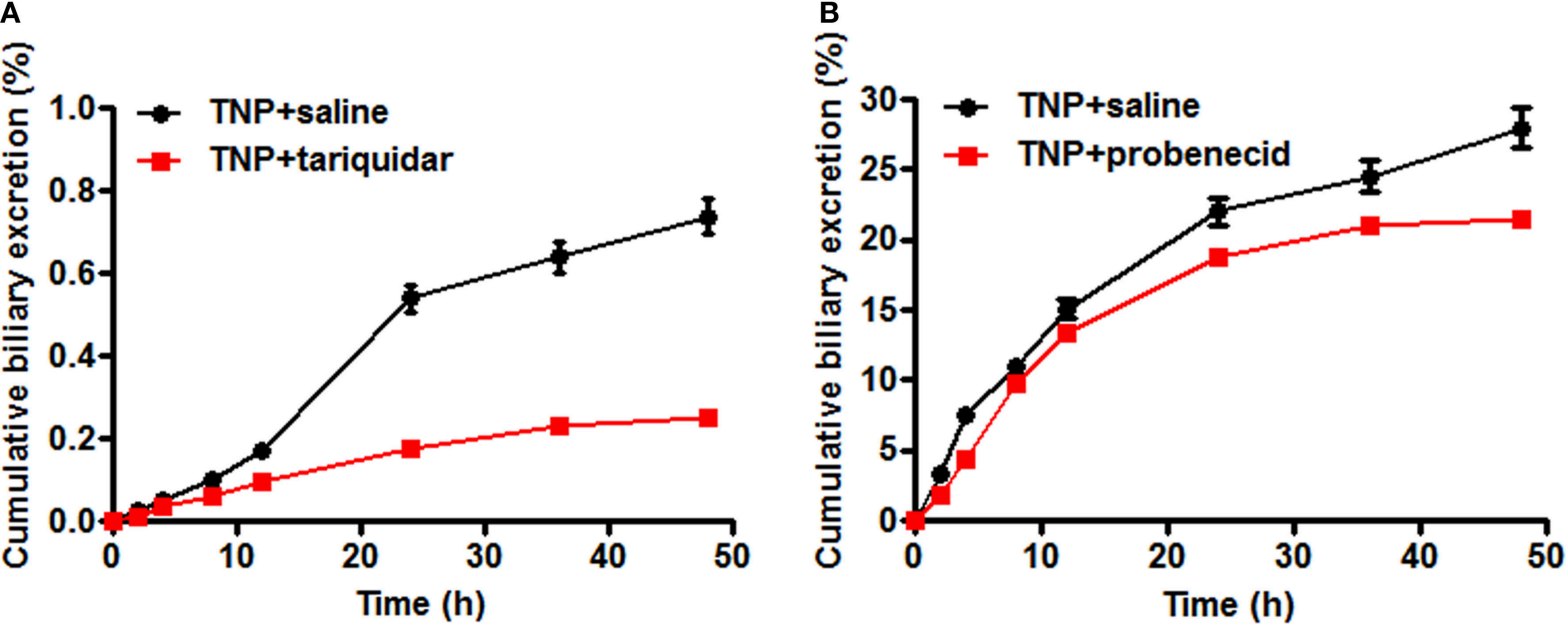
**Effect of tariquidar or probenecid on biliary excretion of TNP and TNP-G in rats**. Tariquidar (10 mg/kg) or probenecid (200 mg/kg) was injected into the caudal vein 15 min prior to the intragastric administration of TNP at a dose of 12 mg/kg. Bile samples were collected at scheduled time intervals. Data are presented as the mean ± SD (*n* = 6). **(A)** The effect of tariquidar on biliary excretion of TNP; **(B)** The effect of probenecid on biliary excretion of TNP-G.

The transporter mediated biliary excretion of TNP and TNP-G was further assessed in BDC rats using transporter inhibitors. As shown in Figure 5, TNP-G was the predominant component detected in the bile of the control group, with a 24 h recovery up to 28% of the TNP dose applied. The biliary excretion of TNP was minor, amounting to less than 1% of the dose. In the group of TNP co-administered with tariquidar (10 mg/kg), the inhibitor significantly decreased the cumulative bile excretion of TNP to 45% of the control level. Similarly, the remarkable reduction of TNP-G biliary excretion by probenecid (200 mg/kg) was observed, with the 24 h excretion rate being decreased to 74% of the control level. In addition, the *in vivo* CL_bile, obs_ was calculated using Equation 5. The CL_bile, obs_ of TNP and TNP-G was 2.4 ± 0.1 and 6.9 ± 0.5 ml/min/kg, respectively, (Supplementary Table 1).

### Effects of chemical inhibitors on TNP and TNP-G pharmacokinetics in rats

**Figure 6:**
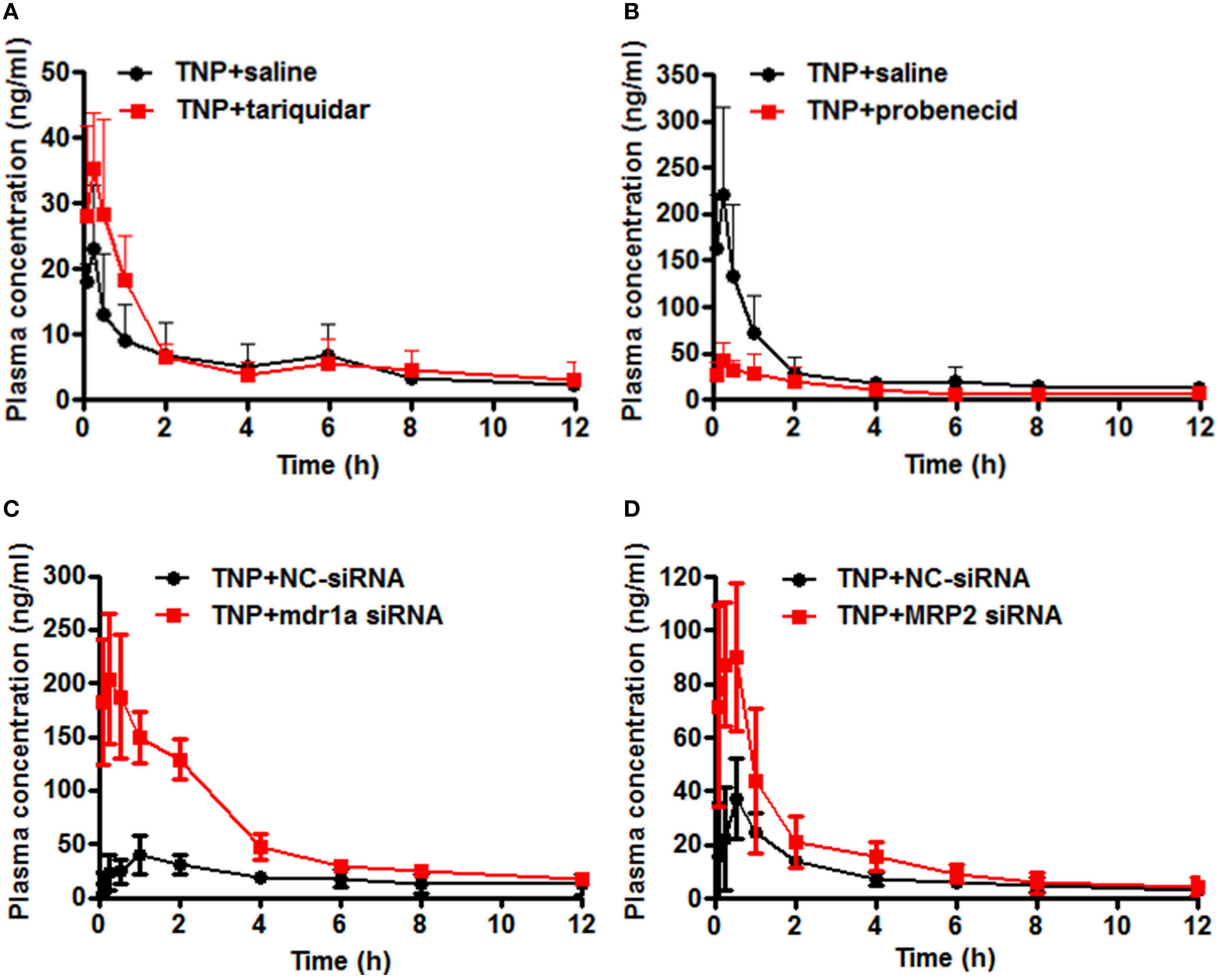
**Effect of chemical inhibitors and siRNA on pharmacokinetics of TNP and TNP-G in rats and mice**. Tariquidar (10 mg/kg) or probenecid (200 mg/kg) was injected into the caudal vein 15 min prior to the intragastric administration of TNP at a dose of 12 mg/kg. siRNA (15 nM) was injected into the caudal vein 2 days prior to the intragastric administration of TNP at a dose of 12 mg/kg. Blood samples were collected at scheduled time intervals. Data are presented as the mean ± SD (*n* = 6). **(A)** The effect of tariquidar on pharmacokinetics of TNP in rats; **(B)** The effect of probenecid on pharmacokinetics of TNP-G in rats; **(C)** The effect of mdr1a siRNA on pharmacokinetics of TNP in mice; **(D)** The effect of MRP2 siRNA on pharmacokinetics of TNP-G in mice.

To evaluate the effect of transporter modulation on TNP pharmacokinetics, the plasma concentration-time curve of TNP and TNP-G was obtained in rats with or without inhibitors (Figures 6A,B). After a single oral dose of 12 mg/kg, the TNP curve for the control group showed a second peak at 6 h, but not for TNP-G. This indicated existing EHC from TNP in rats. When co-administered with tariquidar, the plasma exposure (C_max_ and AUC_0-*t*_) was significantly increased, and the elimination of TNP (T_1/2_ and MRT) was remarkably reduced (Table 2). In contrast, the plasma exposure of TNP-G was remarkably reduced by the concomitant dose with probenecid. The plasma elimination of TNP-G was decreased with increased T_1/2_ and MRT.

**Table 2:** **Pharmacokinetic parameters of TNP and TNP-G after administration of TNP to rats pretreated with tariquidar or probenecid**.

**TNP**	**TNP + saline**	**TNP + probenecid**	**TNP + tariquidar**
T_1/2_ (h)	7.8 ± 1.4	5.6 ± 2.6	3.4 ± 1.7^#^
C_max_ (ng/ml)	16.2 ± 7.8	24.2 ± 3.8	47.3 ± 8.1^#^
AUC_(0-t)_ (ng.h/ml)	38.9 ± 23.8	39.5 ± 16.8	119.0 ± 68.6^#^
MRT (h)	12.2 ± 2.2	7.4 ± 3.9	6.4 ± 3.3^#^
**TNP-G**			
T_1/2_ (h)	6.4 ± 2.7	13.7 ± 5.7^#^	6.3 ± 2.1
C_max_ (ng/ml)	227.7 ± 93.5	43.1 ± 19.3^##^	250.6 ± 52.8
AUC_(0-t)_ (ng.h/ml)	488.5 ± 92.2	294.1 ± 100.1^#^	645.1 ± 117.8
MRT (h)	8.4 ± 3.8	19.6 ± 8.1^#^	7.1 ± 0.9

Probenecid (200 mg/kg), tariquidar (10 mg/kg), and saline (control) was injected into caudal vein 15 min prior to the intragastric administration of TNP at a dose of 12 mg/kg. Data are expressed as mean ± SD (n = 6).

# P < 0.05,

## *P < 0.01 compared with saline group*.

### Effects of siRNA on TNP and TNP-G disposition in mice

To validate the changes observed in rats treated with chemical inhibitors, pharmacokinetics studies in mice treated with siRNA were further conducted. The plasma concentration-time profiles of TNP and TNP-G were shown in Figures 6C,D. The pharmacokinetic parameters were calculated and listed in Table 3. As expected, treatment with the mdr1a siRNA significantly increased the TNP plasma exposure, with the C_max_ increasing from 38.0 ± 12.6 of the control group to 211.9 ± 61.3 ng/ml of the siRNA group and the AUC from 209.64 ± 40.5 to 708.6 ± 108.3 ng·h/ml. In the MRP2 siRNA group, a comparable change was also noted for TNP-G, with C_max_ increasing from 37.1 ± 14.9 to 100.2 ± 17.6 ng/ml and the AUC from 108.4 ± 19.6 to 202.7 ± 70.7 ng·h/ml.

**Table 3:** **Pharmacokinetic parameters of TNP and TNP-G after administration of TNP to mice pretreated with siRNA**.

**TNP**	**TNP + NC-siRNA**	**TNP + mdr1a siRNA**
t_1/2_ (h)	5.1 ± 1.1	5.1 ± 2.1
C_max_ (ng/ml)	38.0 ± 12.6	211.9 ± 61.3^##^
AUC_(0-t)_ (ng.h/ml)	209.64 ± 40.5	708.6 ± 103.8^##^
MRT (h)	4.57 ± 0.46	3.29 ± 0.28^#^
**TNP-G**	**TNP** + **NC-siRNA**	**TNP** + **MRP2 siRNA**
t_1/2_ (h)	4.3 ± 1.7	6.1 ± 1.1
C_max_ (ng/ml)	37.1 ± 14.9	100.2 ± 17.6^##^
AUC_(0-t)_ (ng.h/ml)	108.4 ± 19.6	202.7 ± 70.7^#^
MRT (h)	5.7 ± 2.0	6.7 ± 1.7

siRNA was injected into caudal vein 2 days prior to the intragastric administration of TNP at a dose of 12 mg/kg. Data are expressed as mean ± SD (n = 6).

# P < 0.05,

## *P < 0.01 compared with saline group*.

## Discussion

The drugs currently in clinical use for treating opioid dependence include the full-opioid agonist (methadone), partial-opioid agonist (buprenorphine), and antagonist (naltrexone) (O'Connor and Fiellin, 2000; Johnson et al., 2003). Although these drugs have been proven to be extremely effective in reducing illicit opioid use, they have some drawbacks, such as low oral bioavailability and the potential of dependence. Thus, compounds with a higher oral bioavailability and lower dependence liability would be more useful for the treatment of opioid abuse (Li, 2008). TNP, a new partial opioid agonist, has been selected and developed for the treatment of drug dependence. The obvious advantages of TNP over buprenorphine, more potent, longer acting, higher oral bioavailability, and lower dependence liability, make TNP a promising drug candidate (Yu et al., 2006). Previous studies suggest that the prolonged effect of TNP is likely associated with its pharmacokinetic properties. The elimination of TNP from plasma is slow, as its excretion can last for ~2 weeks (Kong et al., 2007; Deng et al., 2012). Relatively low plasma concentration and slower elimination are considered to be the desired properties for drugs used in the treatment of opioid dependence, thereby reducing the dose frequency and increasing the compliance of patients (Yu et al., 2006; Kong et al., 2007).

In the present study, we present evidence that P-gp and MRP2 are involved in the biliary clearance of TNP and TNP-G. The ATPase assay with transporter membranes indicated that TNP was a P-gp substrate and its TNP-G was a MRP2 substrate. In Caco-2 monolayer transport, P-gp and MRP2 inhibitors cyclosporine A and MK571 increased the apparent permeability of TNP, suggested that it is a substrate of P-gp and MRP2 (Li et al., 2010). To further assess the role of transporters in cellular accumulation, we conducted the uptake experiments in HepG2 cells treated with siRNA. Knockdown of the P-gp gene by mdr1a siRNA significantly increased the intracellular concentration of TNP only, and the MRP2 siRNA treatment solely increased the TNP-G concentration. This results confirmed the findings from ATPase assay.

An *in vitro* study using the SCRH model was then conducted to confirm the transporter mediated canalicular efflux. SCRH forms intact bile canalicular networks and maintains functional expression of the transport proteins for several days (Liu et al., 1999b; Hoffmaster et al., 2004). This model has been widely used to assess drug uptake and efflux, DDIs, and hepatotoxicity (Kimoto et al., 2012; Zhuang et al., 2013). The results of the SCRH study demonstrated that P-gp played significant role in the biliary excretion of TNP. The canalicular efflux of TNP was remarkably affected by P-gp inhibitors or inducers. In the case of TNP-G, MRP2 was responsible for its biliary excretion, and similar effects were observed when SCRH was co-incubated with the MRP2 inhibitor or inducer. The involvement of P-gp and MRP2 in the biliary excretion of TNP and its conjugate was further confirmed in the BDC rats. The cumulative bile excretion of TNP and TNP-G was significantly inhibited by tariquidar and probenecid, respectively.

*In vitro*-*in vivo* extrapolation (IVIVE) by utilizing CL_bile, int_ obtained from SCRH has become a common approach to predict the *in vivo* biliary clearance of drugs (Nakakariya et al., 2012; Zou et al., 2013; Pfeifer et al., 2014). The *in vivo* CL_bile, pred_ of some marketed drugs and new chemical entities predicted from CL_bile, int_ in SCRH or sandwich-cultured human hepatocytes (SCHH) showed a reasonable correlation with the *in vivo* CL_bile, obs_ (Ghibellini et al., 2007; Abe et al., 2008, 2009; Fukuda et al., 2008). In our study, the CL_bile, pred_ values of TNP and TNP-G obtained from SCRH based on the plasma total concentration was ~2- and 1.3-fold, respectively, of the measured CL_bile, obs_ in rats. The CL_bile, pred_ values calculated from Equation (4), by taking into consideration the unbound fraction, were ~7- to 30-fold lower than CL_bile, obs_, which is a reasonable range when compared with the data in the literature (Abe et al., 2008, 2009). The IVIVE using data from the SCRH model in combination with the bile excretion data from BDC rats may provide important information for an improved understanding of the mechanism underlying the hepatobiliary clearance of TNP. However, further studies using SCHH are needed, as some researchers suggested that the SCHH were more suitable for *in vitro* prediction of human clearance (Abe et al., 2008).

The impact of transporter modulation on plasma pharmacokinetics of TNP and TNP-G was further investigated by co-administration of P-gp or MRP2 inhibitors in rats or by pretreatment with siRNA in mice. When compared with the control group, significant increase of TNP plasma exposure was observed in the tariquidar treated group, with a 2.9-fold increase in the Cmax and 3.1-fold increase in the AUC. Treatment of mdr1a siRNA in mice increased Cmax and AUC by 5.6 and 3.4-fold, respectively, as compared to the control group. The remarkably increased Cmax could be associated with the inhibition on P-gp protein allocated at intestinal epithelia (Giacomini et al., 2010). In present study, several folds of Cmax and AUC changes in mice after siRNA treatment also indicated that siRNA not only effected the biliary excretion, but significantly impacted the absorption of TNP. In addition to the changes of TNP plasma exposure, the elimination T_1/2_, and MRT were significantly reduced in both tariquidar and mdr1a siRNA treated group when compared to the control group, indicating that the clearance of the drug was notably increased. This was likely the result of reduced biliary excretion and interrupted EHC of TNP and TNP-G. EHC occurs by bile excretion and intestinal reabsorption of a drug, sometimes with hepatic or intestinal conjugation and intestinal deconjugation (Roberts et al., 2002). EHC changes the pharmacokinetics of drugs by influencing T_1/2_ and AUC (Marier et al., 2002; Xing et al., 2005). Our previous study confirmed, by using a paired rat model, that TNP, and TNP-G underwent significant EHC (Deng et al., 2012). The EHC had a significant effect on the terminal elimination of TNP, resulting in the prolonged retention of the drug in rats. The EHC could be benefit to TNP pharmacological effects. Reduction in biliary excretion and EHC of TNP might impact the duration of drug action. Furthermore, TNP is a dual substrate of metabolizing enzymes and P-gp, which undergoes CYP-mediated oxidative metabolism in the liver (Deng et al., 2010). Modulations of P-gp by tariquidar or mdr1a siRNA may increase the exposure of the drug to the metabolizing enzymes in the liver. The interplay between the drug enzymes and efflux transporter might further complicate the hepatic clearance of TNP.

In contrast to TNP, the alteration of TNP-G disposition was contradictory between probenecid group and siRNA treatment group. In the MRP2 siRNA pretreated mice, the plasma exposure of TNP-G was remarkably increased, with C_max_ and AUC_0-*t*_ being 2.7-and 1.9-fold increased, respectively, when compared to the NC-siRNA group, and the retention time of the drug (MRT) was significantly reduced. This was consistent with the changes observed for TNP. In the probenecid treated group, the T_1/2_ and MRT of TNP-G were prolonged, and the C_max_, and AUC_0-*t*_ were significantly decreased. TNP-G is a glucoronide metabolite of TNP formed by UGT1A1 (Dong et al., 2013). As a chemical inhibitor, probenecid has a dual function on the MRP2 transporter and drug metabolizing enzymes. Several previous studies showed the inhibitory effect of probenecid on UGTs (Miners and Mackenzie, 1991; Uchaipichat et al., 2004). We performed the *in vitro* inhibition experiment using rat liver microsomes and recombinant UGT1A1. However, the results showed that probenecid had no significant effect on TNP glucuronidation (Supplementary Table 4). The formation and disposition of TNP-G *in vivo* is a complex process involving the glucuronidation of TNP in the liver and intestine, hepatobiliary excretion, deconjugation, and reabsorption of the conjugate in the intestine. Interference in each step of aforementioned processes may affect the pharmacokinetic profile of TNP-G. Further investigations are warranted to understand the mechanisms underlining the interaction between probenecid and TNP-G.

## Conclusion

The present study demonstrated the major roles of P-gp and MRP2 in biliary excretion of TNP and TNP-G, respectively. Inhibition of P-gp or MRP2 using a chemical inhibitor or siRNA significantly affected the bile excretion of TNP or TNP-G and subsequently caused the alteration in pharmacokinetics of the drug. The potential DDIs of TNP with P-gp or MRP2 modulators should be considered in the treatment of opioid dependence.

## Author contributions

Participated in research design: LK, XZ, and HL. Conducted experiments: LK, GS, ZW, WX, and MY. Performed data analysis: LK, ZG, and XZ. Wrote or contributed to the writing of the manuscript: LK, GS, and HL. Project manager: ZW.

### Conflict of interest statement

The authors declare that the research was conducted in the absence of any commercial or financial relationships that could be construed as a potential conflict of interest.

## Abbreviations

TNP: thienorphine
TNP-G: thienorphine glucuronide conjugate
EHC: enterohepatic circulation
MDR1: multidrug resistance protein 1
P-gp: P-glycoprotein
MRP2: multidrug resistance-associated protein 2
BCRP: breast cancer resistance protein
DDIs: drug-drug interactions
BDC: bile-duct cannulated
SCRH: sandwich-cultured rat hepatocyte
LC-MS/MS: liquid chromatography coupled to tandem mass spectrometry
BEI: biliary excretion index
AUC: area under the plasma concentration time curve
MRT: mean resident time.

## References

[B1] Abe K. Bridges A. S. Brouwer K. L. (2009). Use of sandwich-cultured human hepatocytes to predict biliary clearance of angiotensin II receptor blockers and HMG-CoA reductase inhibitors. Drug Metab. Dispos. 37, 447–452. 10.1124/dmd.108.023465 19074974 PMC2680516

[B2] Abe K. Bridges A. S. Yue W. Brouwer K. L. (2008). *In vitro* biliary clearance of angiotensin ii receptor blockers and 3-Hydroxy-3-methylglutaryl-Coenzyme a reductase inhibitors in sandwich-cultured rat hepatocytes: comparison with i*n vivo* Biliary Clearance. J. Pharmacol. Exp. Ther. 326, 983–990. 10.1124/jpet.108.138073 18574002 PMC2581923

[B3] Bi Y. A. Kazolias D. Duignan D. B. (2006). Use of cryopreserved human hepatocytes in sandwich culture to measure hepatobiliary transport. Drug Metab. Dispos. 34, 1658–1665. 10.1124/dmd.105.009118 16782767

[B4] Deng J. T. Zhuang X. M. Li H. (2010). *In vitro* comparison of thienorphine metabolism in liver microsomes of human, Beagle dog and rat. Yao Xue Xue Bao. 45, 98–103. 10.16438/j.0513-4870.2010.01.012 21351457

[B5] Deng J. T. Zhuang X. M. Shen G. L. Li H. Gong Z. H. (2012). Biliary excretion and enterohepatic circulation of thienorphine and its glucuronide conjugate in rats. Acta Pharm. Sin. B 2, 172–178. 10.1016/j.apsb.2012.02.007

[B6] Dong R. H. Fang Z. Z. Zhu L. L. Ge G. B. Li X. B. Hu C. M. (2013). Deep understanding of the interaction between thienorphine and UDP-glucuronosyltransferase (UGT) isoforms. Xenobiotica 43, 133–139. 10.3109/00498254.2012.706723 22813462

[B7] Fukuda H. Ohashi R. Tsuda-Tsukimoto M. Tamai I. (2008). Effect of plasma protein binding on *in vitro*-*in vivo* correlation of biliary excretion of drugs evaluated by sandwich-cultured rat hepatocytes. Drug Metab. Dispos. 36, 1275–1282. 10.1124/dmd.107.019026 18388177

[B8] Ghibellini G. Vasist L. S. Leslie E. M. Heizer W. D. Kowalsky R. J. Calvo B. F.. (2007). *In vitro*-*in vivo* correlation of hepatobiliary drug clearance in humans. Clin. Pharmacol. Ther. 81, 406–413. 10.1038/sj.clpt.6100059 17235333

[B9] Giacomini K. M. Huang S. M. Tweedie D. J. Benet L. Z. Brouwer K. L. Chu X.. (2010). Membrane transporters in drug development. Nat. Rev. Drug Discov. 9, 215–236. 10.1038/nrd3028 20190787 PMC3326076

[B10] Hoffmaster K. A. Turncliff R. Z. LeCluyse E. L. Kim R. B. Meier P. J. Brouwer K. L. (2004). P-glycoprotein expression, localization, and function in sandwich-cultured primary rat and human hepatocytes: relevance to the hepatobiliary disposition of a model opioid peptide. Pharm. Res. 21, 1294–1302. 10.1023/B:PHAM.0000033018.97745.0d 15290872

[B11] Johnson R. E. Strain E. C. Amass L. (2003). Buprenorphine: how to use it right. Drug Alcohol. Depend. 70, S59–S77. 10.1016/s0376-8716(03)00060-7 12738351

[B12] Kimoto E. Walsky R. Zhang H. Bi Y. A. Whalen K. M. Yang Y. S.. (2012). Differential modulation of cytochrome P450 activity and the effect of 1-aminobenzotriazole on hepatic transport in sandwich-cultured human hepatocytes. Drug Metab. Dispos. 40, 407–411. 10.1124/dmd.111.039297 22031626

[B13] Kong L. L. Yang H. Y. Yuan M. Zhuang X. M. Li H. (2015b). The evaluation of efflux transporter model based on RNA interference technology *in vitro*. Yao Xue Xue Bao 50, 1122–1127. 10.16438/j.0513-4870.2015.09.015 26757548

[B14] Kong L. L. Zhuang X. M. Yang H. Y. Yuan M. Xu L. Li H. (2015a). Inhibition of P-glycoprotein gene expression and function enhances triptolide-induced hepatotoxicity in mice. Sci. Rep. 5:11747. 10.1038/srep11747 26134275 PMC4488747

[B15] Kong Q. Qiao J. Z. Wang X. Y. Yuan S. Zhang Z. Q. Gong Z. H.. (2007). Simultaneous determination of thienorphine and its active metabolite thienorphine-glucuronide in rat plasma by liquid chromatography–tandem mass spectrometry and its application to pharmacokinetic studies. J. Chromatogr. B Analyt. Technol. Biomed. Life. Sci. 859, 52–61. 10.1016/j.jchromb.2007.09.028 17933594

[B16] Li J. (2008). Recent progress in the research field of neuropharmacology in China. Cell Mol. Neurobiol. 28, 185–204. 10.1007/s10571-007-9252-z 18240016 PMC11515025

[B17] Li J. X. Becker G. L. Traynor J. R. Gong Z. H. France C. P. (2007). Thienorphine: receptor binding and behavioral effects in rhesus monkeys. J. Pharmacol. Exp. Ther. 321, 227–236. 10.1124/jpet.106.113290 17220427

[B18] Li Z. Zhuang X. M. Li S. Y. Zhang Z. Q. Ruan J. X. (2010). Transport of thiophenorphine across Caco-2monolayer model. Chin. J. Pharmacol. Toxicol. 24, 64–68. 10.3867/j.issn.1000-3002.2010.01.011

[B19] Liu H. Zhong B. H. Liu C. H. Wu B. Gong Z. H. (2005). Synthesis, crystal structure and pharmacological study of N-cyclopropylmethyl-7a- [1-(R)-1-hydroxy-1-methyl-3-(thien-2-yl)propyl]-6,14-endo-ethanotetrahydro-oripavine. *Acta. Chim*. Slov. 52, 80–85.

[B20] Liu X. Chism J. P. LeCluyse E. L. Brouwer K. R. Brouwer K. L. (1999a). Correlation of biliary excretion in sandwich-cultured rat hepatocytes and *in vivo* in rats. *Drug Metab*. Dispos. 27, 637–644. 10348791

[B21] Liu X. LeCluyse E. L. Brouwer K. R. Gan L. S. Lemasters J. J. Stieger B.. (1999b). Biliary excretion in primary rat hepatocytes cultured in a collagen-sandwich configuration. *Am. J*. Physiol. 277, G12–G21. 10409146 10.1152/ajpgi.1999.277.1.G12

[B22] Marier J. F. Vachon P. Gritsas A. Zhang J. Moreau J. P. Ducharme M. P. (2002). Metabolism and disposition of resveratrol in rats: extent of absorption, glucuronidation, and enterohepatic recirculation evidenced by a linked-rat model. J. Pharmacol. Exp. Ther. 302, 369–373. 10.1124/jpet.102.033340 12065739

[B23] Miners J. O. Mackenzie P. I. (1991). Drug glucuronidation in humans. Pharmacol. Ther. 51, 347–369. 10.1016/0163-7258(91)90065-T 1792239

[B24] Nakakariya M. Ono M. Amano N. Moriwaki T. Maeda K. Sugiyama Y. (2012). *In vivo* biliary clearance should be predicted by intrinsic biliary clearance in sandwich-cultured hepatocytes. Drug Metab. Dispos. 40, 602–609. 10.1124/dmd.111.042101 22190695

[B25] O'Connor P. G. Fiellin D. A. (2000). Pharmacologic treatment of heroin dependent patients. *Ann. Intern*. Med. 133, 40–54. 10.7326/0003-4819-133-1-200007040-00008 10877739

[B26] Pfeifer N. D. Hardwick R. N. Brouwer K. L. (2014). Role of hepatic efflux transporters in regulating systemic and hepatocyte exposure to xenobiotics. Annu. Rev. Pharmacol. Toxicol. 54, 509–535. 10.1146/annurev-pharmtox-011613-140021 24160696

[B27] Roberts M. S. Magnusson B. M. Burczynski F. J. Weiss M. (2002). Enterohepatic circulation: physiological, pharmacokinetic and clinical implications. Clin. Pharmacokinet. 41, 751–790. 10.2165/00003088-200241100-00005 12162761

[B28] Sai Y. (2005). Biochemical and molecular pharmacological aspects of transporters as determinants of drug disposition. Drug Metab. Pharmacokinet. 20, 91–99. 10.2133/dmpk.20.91 15855719

[B29] Shen G. Zhuang X. Xiao W. Kong L. Tan Y. Li H. (2014). Role of CYP3A in regulating hepatic clearance and hepatotoxicity of triptolide in rat liver microsomes and sandwich-cultured hepatocytes. Food Chem. Toxicol. 71, 90–96. 10.1016/j.fct.2014.05.020 24910460

[B30] Shitara Y. Sugiyama Y. (2006). Pharmacokinetic and pharmacodynamic alterations of 3-hydroxy-3-methylglutaryl coenzyme A (HMG-CoA) reductase inhibitors: drug-drug interactions and interindividual differences in transporter and metabolic enzyme functions. Pharmacol. Ther. 112, 71–105. 10.1016/j.pharmthera.2006.03.003 16714062

[B31] Tchaparian E. H. Houghton J. S. Uyeda C. Grillo M. P. Jin L. (2011). Effect of culture time on the basal expression levels of drug transporters in sandwich-cultured primary rat hepatocytes. Drug Metab. Dispos. 39, 2387–2394. 10.1124/dmd.111.039545 21865320 PMC3226373

[B32] Uchaipichat V. Mackenzie P. I. Guo X. H. Gardner-Stephen D. Galetin A. Houston J. B.. (2004). Human udp-glucuronosyltransferases: isoform selectivity and kinetics of 4-methylumbelliferone and 1-naphthol glucuronidation, effects of organic solvents, and inhibition by diclofenac and probenecid. Drug Metab. Dispos. 32, 413–423. 10.1124/dmd.32.4.413 15039294

[B33] Xing J. Chen X. Zhong D. (2005). Absorption and enterohepatic circulation of baicalin in rats. Life Sci. 78, 140–146. 10.1016/j.lfs.2005.04.072 16107266

[B34] Yu G. Li S. H. Cui M. X. Yan L. D. Yong Z. Zhou P. L.. (2013). Multiple mechanisms underlying the long duration of action of thienorphine, a novel partial opioid agonist for the treatment of addiction. CNS Neurosci. Ther. 20, 282–288. 10.1111/cns.12210 24330593 PMC6492997

[B35] Yu G. Yue Y. J. Cui M. X. Gong Z. H. (2006). Thienorphine is a potent long-acting partial opioid agonist: a comparative study with buprenorphine. J. Pharmacol. Exp. Ther. 318, 282–287. 10.1124/jpet.105.099937 16569757

[B36] Zhuang X. M. Shen G. L. Xiao W. B. Tan Y. Lu C. Li H. (2013). Assessment of the roles of P-glycoprotein and cytochrome P450 in triptolide-induced liver toxicity in sandwich-cultured rat hepatocyte model. Drug Metab. Dispos. 41, 2158–2165. 10.1124/dmd.113.054056 24065861

[B37] Zou P. Liu X. Wong S. Feng M. R. Liederer B. M. (2013). Comparison of *in vitro*-*in vivo* extrapolation of biliary clearance using an empirical scaling factor versus transport-based scaling factors in sandwich-cultured rat hepatocytes. J. Pharm. Sci. 102, 2837–2850. 10.1002/jps.23620 23712819

