# Supplementary material for: Inhibition of P-Glycoprotein and Multidrug Resistance-Associated Protein 2 Regulates the Hepatobiliary Excretion and Plasma Exposure of Thienorphine and Its Glucuronide Conjugate
Source: Front Pharmacol. 2016 Aug 9;7:242. doi: 10.3389/fphar.2016.00242 (PMC4977286; doi:10.3389/fphar.2016.00242)
Supplement: Supplementary file 1 [file Table1.DOC]

**Table 1**

Observed *in vivo* CL_bile,obs_ and predicted CL_bile,pred_ based on *in vitro* CL_bile, int_

| Compound | CL_bile, int_  (ml/min/kg) |  | CL_bile,pred_  (ml/min/kg) | | CL_bile,obs_  (ml/min/kg) |
| --- | --- | --- | --- | --- | --- |
|  | From Eq. 2 | f_u,p_ | From Eq. 3 | From Eq. 4 | From Eq.5 |
| TNP | 5.8±0.5 | 0.05 | 5.0±0.4 | 0.3±0.02 | 2.4±0.1 |
| TNP-G | 11.2±2.7 | 0.02 | 8.7±1.7 | 0.2±0.01 | 6.9±0.5 |

CL_bile,int_ was calculated from Eq.2, predicted CL_bile,pred_ was calculated from Eq.3. Taking into consideration the plasma unbound fraction (f_u,p_), predicted CL_bile,pred_ was calculated according to Eq.4. f_u,p_ of TNP and TNP-G was investigated in previous study. *In vivo* CL_bile,obs_ was calculated from Eq.5 according to present results. Data are presented as mean ± SD (n=3).
