# Supplementary material for: Inhibition of P-Glycoprotein and Multidrug Resistance-Associated Protein 2 Regulates the Hepatobiliary Excretion and Plasma Exposure of Thienorphine and Its Glucuronide Conjugate
Source: Front Pharmacol. 2016 Aug 9;7:242. doi: 10.3389/fphar.2016.00242 (PMC4977286; doi:10.3389/fphar.2016.00242)
Supplement: Supplementary file 2 [file Table2.DOC]

**Table 2**

CL_bile,int_ of TNP with or without P-gp inhibitors and inducer in SCRH

| Inhibitors | | | | Inducer | |
| --- | --- | --- | --- | --- | --- |
| Tariquidar  (μM) | CL_bile,int_  (ml/min/kg) | Verapamil  (μM) | CL_bile,int_  (ml/min/kg) | Quercetin  (μM) | CL_bile,int_  (ml/min/kg) |
| 0 | 16.5±1.0 | 0 | 12.9±1.7 | 0 | 11.7±1.0 |
| 0.5 | 9.2±0.1^##^ | 10 | 5.9±0.8^##^ | 3 | 15.6±5.6 |
| 2.5 | 5.1±1.4^###^ | 50 | 5.2±1.5^##^ | 15 | 18.7±0.6^##^ |
| 5.0 | 2.8±1.0^###^ | 100 | 3.4±0.4^###^ | 30 | 20.4±0.5^###^ |

The rat hepatocytes were cultured for 5 days prior to the study, then tariquidar and verapamil were added 30 min and quercetin was added 3 days prior to TNP. Data are expressed as mean±SD (n=3). ^##^*P*<0.01, ^###^*P*<0.001 compared with control group without inhibitor.
