# Supplementary material for: Inhibition of P-Glycoprotein and Multidrug Resistance-Associated Protein 2 Regulates the Hepatobiliary Excretion and Plasma Exposure of Thienorphine and Its Glucuronide Conjugate
Source: Front Pharmacol. 2016 Aug 9;7:242. doi: 10.3389/fphar.2016.00242 (PMC4977286; doi:10.3389/fphar.2016.00242)
Supplement: Supplementary file 3 [file Table3.DOC]

**Table 3**

CL_bile,int_ of TNP-G with or without MRP2 inhibitors and inducer in SCRH

| Dose  (μM) | CL_bile,int_ (ml/min/kg) | | |
| --- | --- | --- | --- |
|  | Inhibitors | | Inducer |
|  | Probenecid | Methotrexate | Dexamethasone |
| 0 | 1.6±0.03 | 3.1±0.4 | 4.2±0.1 |
| 10 | 0.8±0.3^#^ | 2.2±0.7 | 5.5±1.0 |
| 50 | 0.7±0.1^##^ | 1.1±0.1^##^ | 8.9±0.3^##^ |
| 100 | 0.3±0.1^###^ | 0.8±0.2^###^ | 11.1±0.1^###^ |

The rat hepatocytes were cultured for 5 days prior to the study, then probenecid and methotrexate were added 30 min and dexamethasone was added 3 days prior to TNP-G. Data are expressed as mean±SD (n=3).  ^#^*P*<0.05, ^##^*P*<0.01, ^###^*P*<0.001 compared with control group without inhibitor.
