# Supplementary material for: Inhibition of P-Glycoprotein and Multidrug Resistance-Associated Protein 2 Regulates the Hepatobiliary Excretion and Plasma Exposure of Thienorphine and Its Glucuronide Conjugate
Source: Front Pharmacol. 2016 Aug 9;7:242. doi: 10.3389/fphar.2016.00242 (PMC4977286; doi:10.3389/fphar.2016.00242)
Supplement: Supplementary file 4 [file Table4.DOC]

**Table 4**

Effect of inhibitors and inducers on glucuronidation of TNP

| Group | TNP-G formation (ng/ml) | |
| --- | --- | --- |
|  | Liver microsome | UGT1A1 |
| Control | 2303.5±107.1 | 1624.1±58.3 |
| Probenecid (100 µM) | 2191.5±123.1 | 1527.5±83.9 |
| Tariquidar (100 µM) | 2154.6±26.9 | 1533.8±25.4 |
| Dexamethasone (100 µM) | 2219.3±52.8 | 1542.4±27.9 |
| Quercetin (100 µM) | 2170.7±33.6 | 1590.8±30.5 |

TNP (5µM) and inhibitors or inducers were incubated with liver microsome and UGT1A1 at 37 °C for 60 min. Data are expressed as mean±SD (n=3).
